# Utility of the REBA MTB-rifa® assay for rapid detection of rifampicin resistant *Mycobacterium Tuberculosis*

**DOI:** 10.1186/1471-2334-13-478

**Published:** 2013-10-15

**Authors:** Eunjin Cho, Isdore Chola Shamputa, Hyun-Kyung Kwak, Jiim Lee, Myungsun Lee, Soohee Hwang, Doosoo Jeon, Cheon Tae Kim, Sangnae Cho, Laura E Via, Clifton E Barry 3rd, Jong Seok Lee

**Affiliations:** 1 Section of Microbiology, International Tuberculosis Research Center, Changwon 475-1, Republic of Korea; 2 Department of Microbiology, Yonsei University College of Medicine, Seoul, Korea; 3 Masan National Hospital, Changwon 475-1, Korea; 4 Tuberculosis Research Section, Laboratory of Clinical Infectious Disease, National Institute of Allergy and Infectious Diseases, National Institutes of Health, Bethesda, MD 20892, USA

**Keywords:** *Mycobacterium tuberculosis*, Rifampicin resistance, REBA MTB-Rifa® assay, *rpoB*

## Abstract

**Background:**

Drug-resistant tuberculosis (TB), including resistance to both rifampicin (RIF) and isoniazid (INH) referred to as multidrug-resistant tuberculosis (MDR-TB), has become an increasing global threat in recent years. Effective management of patients infected with MDR-TB strains requires identifying such patients by performing conventional drug-susceptibility testing (DST) on bacteria isolated from sputum, a process that can take up to 2 months. This delay in diagnosis can result in worsening and continued transmission of MDR-TB. Molecular methods that rely upon nucleic acid amplification of specific alleles known to be associated with resistance to specific drugs have been helpful in shortening the time to detect drug resistant TB.

**Methods:**

We investigated the utility of the REBA MTB-Rifa®, a commercially available line probe assay (LPA) for detecting rifampicin (RIF) resistance in the RIF resistance-determining region (RRDR) of the *rpoB* gene. Altogether, 492 *Mycobacterium tuberculosis* (*M. tuberculosis*) clinical isolates and additional 228 smear- and culture-positive sputum samples with confirmed *M. tuberculosis* were collected from subjects with suspected MDR-TB in South Korea. The results were compared with conventional phenotypic DST and sequencing of the *rpoB* gene.

**Results:**

A total of 215 of the 492 isolates were resistant to RIF by conventional DST, and of which 92.1% (198/215) were MDR-TB strains. The REBA MTB-Rifa® assay identified RIF resistance in 98.1% (211/215) of these isolates but failed to identify resistance in four phenotypically RIF resistant isolates. These four isolates lacked mutations in the RRDR but three were confirmed to be MDR-TB strains by sequencing. The sensitivity and specificity of this test for clinical isolates was thus 98.1% (211/215) and 100% (277/277), respectively. When applied directly to 228 smear positive sputum samples, the sensitivity and the specificity of REBA MTB-Rifa® assay was 100% (96/96, 132/132), respectively.

**Conclusions:**

These findings support the use of the REBA MTB-Rifa® assay for rapid detection of RIF resistance on clinical isolates and smear positive sputum samples. The results also suggest that RIF resistance is a good surrogate marker of MDR-TB in South Korea and the need to add more probes to other LPAs which can cover newly identified mutations relevant to RIF resistance.

## Background

Drug-resistant tuberculosis (TB) has become an increasing global threat in recent years. The World Health Organization has estimated that the global burden of multidrug-resistant TB (MDR-TB, defined as combined resistance to INH and RIF) will increase from the current 500,000 cases per year to nearly 2,000,000 cases by 2015 [1]. Expanding efforts to treat patients infected with MDR-TB is leading to the generation of extensively drug-resistant TB (XDR-TB, defined as MDR-TB plus resistant to any fluoroquinolone and at least one of three second-line anti-TB injectable drugs). Treating patients with MDR-TB requires identifying them by performing DST on bacteria isolated from sputum, a process that can take up to 2 months. This delay in diagnosis can result in worsening of disease and further transmission of MDR-TB disease.

To avoid this delay in proper diagnosis, several rapid diagnostic methods have been developed that rely on amplification of specific alleles known to be associated with resistance to specific drugs. LPA, a method that is based on nucleic acid amplification followed by hybridization of amplicons to target probes immobilized on membranes to detect resistance-associated sequence polymorphisms, are widely used [2]. LPAs have been officially endorsed by the WHO to detect MDR-TB from cultured isolates and directly from smear positive sputum samples [3]. Even though LPAs, and other molecular techniques are very accurate and rapid in detecting mutations, they are intrinsically limited to known mutations in drug targets. For RIF, the diagnostic accuracy of detection is quite robust and up to 97% of isolates can be correctly identified as resistant or susceptible [4]. Although LPAs typically includes both wild type and mutant alleles, these must be targeted to relatively short DNA segments that have been demonstrated to be involved in resistance to specific agents. Newer ways of detecting RIF resistance alleles, such as the recently introduced GeneXpert MTB/RIF (Cepheid, Sunnyvale, CA) [5] are also limited by knowledge of the resistance mechanisms involved suggesting detection of new alleles associated with resistance is therefore an important objective.

The widespread use of RIF in global TB control programs has inevitably given rise to resistance. The target of RIF is the β-subunit of the bacterial DNA-dependent RNA polymerase, which is encoded by the *rpoB* gene. In the majority of the RIF resistant isolates, mutations are found within an 81-bp region known as the RIF resistance determining region or RRDR. This region encodes 27 amino acids and corresponds to codons 507 to 533. Mutations within this region account for up to 95% of the RIF resistance observed [6-8]. INH has a more complex mode of action and resistance occurs through several discrete mechanisms involving many genes, therefore detection of INH resistance through molecular tests is less reliable than RIF [9,10]. RIF resistance generally occurs in combination with INH and therefore RIF resistance has been proposed as a surrogate maker for MDR-TB [11,12]. Despite the simplicity and utility of self-contained diagnostic tests such as the GeneXpert system, these are twice as expensive as LPAs in a country like Korea that does not qualify for preferential pricing considerations. Since a large fraction of Korean TB patients are indigent the price of such testing can place them out of reach. In addition, reengineering cartridges to take advantage of newly discovered resistance alleles is not trivial [13]. In settings where sufficient technical and microbiological expertise is not limiting, and self-contained molecular tests are expensive, LPAs offer an affordable, accurate and flexible alternative.

In this study, we evaluated the utility of reverse blot assay (REBA MTB-Rifa®) for rapid detection of RIF resistance and assessed its ability to predict RIF resistance for MDR-TB in South Korea using both *M. tuberculosis* isolates and clinical specimens in comparison with phenotypic DST, and *rpoB* gene sequencing results.

## Methods

### Samples

**Figure 1:**
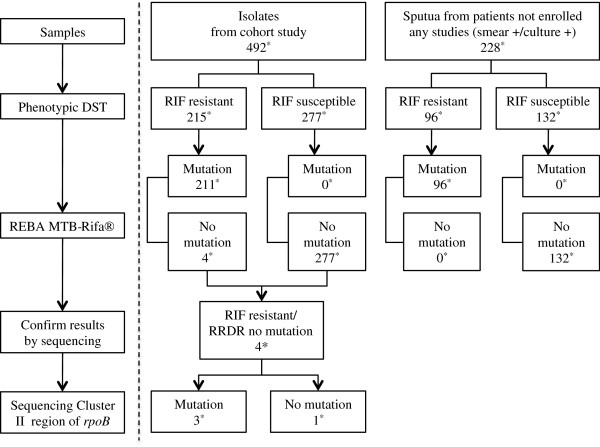
**Schema of the study design.** *, Number of samples; RIF, rifampicin; RRDR, rifampicin resistance determining region.

A total of 492 *M. tuberculosis* isolates isolated from sputa of all consecutive patients with active pulmonary TB at Masan National Hospital (NMH) in South Korea who were enrolled in a prospective observational cohort study (ClinicalTrials.gov identification number, NCT00341601) between 2005 and 2008 were included in the study. The study was reviewed and approved by both the NMH and National Institute of Allergy and Infectious Diseases (NIAID) institutional ethics review boards and all participants gave written informed consent. These isolates were included in the study without regard to their drug susceptibility [14]. Separate from these isolates, 228 recent acid-fast bacilli positive sputum samples with *M. tuberculosis* culture results from routine clinical testing were also included (Figure 1).

### Clinical isolates and drug susceptibility testing

Sputum specimens obtained from each participant were processed using sodium hydroxide and N-acetyl-L-cysteine and then screened for acid-fast bacilli using the *Ziehl-Neelsen* (ZN) staining method and cultured in Ogawa egg slants. DST was performed on Lowenstein-Jensen egg slants containing the relevant anti-TB drugs according to WHO recommendation [15]. Isolates were tested for resistance to critical concentrations of INH (0.2 μg/mL), RIF (40 μg/mL), EMB (2.0 μg/mL), PZA (100 μg/mL, Wayne’s pyrazinamidase assay [16]), ofloxacin (2 μg/mL), and kanamycin (40 μg/mL). *M. tuberculosis* H37Rv (ATCC 27294) was used as a positive control in all experiments.

### Minimum inhibitory concentration (MIC) determination

A 96-well microtiter plate containing RIF at concentrations ranging from 0.25 μg/mL to 64 μg/mL was used to test each sample. Colonies were scraped from a 7H10 agar plate and vortexed in a sterile tube containing 7H9 media with Tween80 and glass beads. The bacterial suspension was allowed to stand for 20 min after adjust the suspension equivalent to McFarland turbidity standard 0.5. From the supernatant, 100 μL of bacterial suspension was inoculated to each well containing RIF on 96-well plates. Perimeter wells on each plate were filled with sterile water to avoid dehydration of the medium during incubation. A growth control without RIF and a sterile control without *M. tuberculosis* strains were also included for each isolate. The plates were sealed and incubated at 37°C in 5% CO_2_ for 7 days. Thereafter 100 uL of Alamar blue (Invitrogen, Carlsbad, USA) was added, the samples were re-incubated and read color changes after 24 hrs. Color change from blue to pink indicated growth of the bacteria, and the MIC was determined as the lowest drug concentration that blue color did not turn to pink [17].

### Isolation of DNA from clinical MTB isolates

A loopful of bacteria from the Ogawa slant culture was suspended in 500 μL of distilled water, and incubated for 10 min at 99°C to sterilize bacteria. After centrifugation for 5 min at 13,000 × g, the supernatant was recovered and stored at −20°C before use.

### DNA preparation from sputum specimens

Sputum specimens were processed using sodium hydroxide and N-acetyl-L-cysteine, and then screened for acid-fast bacilli with the ZN staining method. Each 500 μL of decontaminated and processed smear positive sample was inoculated into liquid culture media (MB/BacT 240). Cultured organisms were sterilized by heating at 99°C for 10 min, and 500 μL was transferred to a microcentrifuge tube that contained 200 μL of 0.1 mm glass beads (Biospec products). The tube was processed in a bead-beater (Biospec products) for 5 min and then centrifuged at 13,000 × g for 5 min. The supernatant were recovered and stored in −20°C before use.

### REBA MTB-Rifa® assay

REBA MTB-Rifa® (YD Diagnostics, Yongin-si, South Korea) [18] is a commercially available LPA based on the reverse hybridization principle that specific oligonucleotide probes are immobilized on a membrane and hybridized under strictly controlled conditions with the biotin-labeled PCR product. It consist of 8 probes, of which five probes representing wild type *rpoB* sequences that span the RRDR i.e. 509-514(WT1), 515-520(WT2), 521-525(WT3), 524-529(WT4) and 530-534(WT5), and three probes [516GAC-GTC(M1), 531TCG-TTG(M2) and 533CTG-CCG(M3)] that account for the most common specific mutations. The assay was performed according to the manufacturer’s instruction. Briefly, genomic DNA from each sample was amplified using two 5′ biotinylated primers with the indicated sequences, *rpoB* 5′long (5′-TCA AGG AGA AGC GCT ACG ACC TGG C-3′) and TR8 long (5′-ACG GGT GCA CGT CGC GGA CCT CCA-3′), to get a 536-bp fragment of *rpoB*. PCR was carried out for 35 cycles using ABI 2720 thermal cycler, with each cycle consist of denaturation at 94°C for 1 min, annealing at 62°C for 30 s, and primer extension at 72°C for 1 min 30 s, followed by a final cycle of 10 min at 72°C. For hybridization, 45 μL of the amplified PCR product was diluted in 105 μL of 2 × SSPE-0.1% SDS and heat denatured at 99°C for 10 min. Denatured DNAs were transferred into parallel channels according to the manufacturer’s instructions (MN45; Immunetics, Cambridge, Mass.) perpendicular to the rows containing oligonucleotide probes and hybridized for 60 min at 50°C. After hybridization, the membrane was washed twice in 250 mL of 2 × saline-sodium phosphate- ethylenediaminetetraacetic acid (SSPE)-0.5% sodium dodecyl sulfate (SDS) buffer at 60°C for 15 min each. The membrane was incubated in 1:2,000-diluted streptavidin-AP conjugate (Roche, 11 093 266 910) for 40 min at 42°C. It was followed by wash twice in 250 mL of 2 × SSPE-0.5% SDS at 42°C, for 10 min each time, and rinsed with 250 mL of 2 × SSPE for 5 min at ambient temperature. Signal was detected by add sufficient amount of chemiluminescent CDP-Star (Amersham, RPN3682) to the membrane and incubate for 5 min, which followed by exposure to X-ray film (Hyperfilm ECL; Amersham) in accordance with the manufacturer’s instructions.

### PCR amplification and sequencing of the products

Twenty microlitres of each DNA was added to the PCR mixture. Two primers with the sequences, TR-8 (5′-TGC ACG TCG CGG ACC TCC A-3′) and TR-9 (5′-TCG CCG CGA TCA AGG AGT-3′) were used to amplify a 187-bp fragment of the RRDR in *rpoB* gene. After the reaction mixtures were incubated at 94°C for 10 min, PCR was carried out in ABI 2720 thermal cycler for 35 cycles, with each cycle consisting of denaturation at 94°C for 60 s, annealing at 62°C for 30 s, and primer extension at 72°C for 90 s, and a final extension step of 10 min at 72°C. To confirm the PCR amplification, 5 μL of PCR products were analyzed in 2% Tris Borate EDTA (TBE) agarose gel electrophoresis. Direct sequencing of PCR products were carried out by Genotech (Daejeon, South Korea) and sequencing results were analyzed using CLC Main Workbench (CLC bio, Aarhus, Denmark).

## Results

### Drug susceptibility patterns of MTB isolates

Out of 492 isolates, 200 isolates were pan-susceptible to all four first line TB drugs (INH, RIF, EMB and PZA) while the remaining 292 isolates were resistant to one or more TB drugs. From 292 drug resistant isolates, 215 isolates were RIF resistant, but only 17 samples were RIF mono-resistant. The remaining 198 (92.1%) isolates were also resistant to INH and therefore MDR-TB strains. Of these 198 MDR-TB isolates, 173 (87.4%) showed further resistance to at least one more first-line drug and 82 (41.4%) isolates were resistant to all tested first line drugs. Among the new cases, 45 cases were resistant to more than one drug of which 10 (5.9%) were MDR-TB but not XDR-TB. Among previously treated cases, 188 (58.4%) cases were MDR-TB of which 35 (18.6%) cases of XDR-TB (data not shown).

### Detection of Rif resistance using the REBA MTB-Rifa® assay on DNA from cultured samples

Alterations in the RRDR were detected in 211 isolates out of 492 samples which tested by REBA MTB-Rifa® assay. All 211 isolates identified by the REBA MTB-Rifa® as having mutations in the RRDR were resistant to RIF by conventional DST testing. However, the REBA MTB-Rifa® failed to identify four isolates that were phenotypic RIF-resistant, and the sensitivity and the specificity of the assay for clinical isolates were 211/215 (98.1%; 95% CI 95.3, 99.5) and 277/277 (100%; 95% CI 98.7, 100), respectively (Table 1). All of the mutations in the RRDR observed by REBA MTB-Rifa® assay were confirmed by DNA sequencing. Of the four phenotypic rifampicin resistant isolates that had no mutations in the RRDR, two harbored mutations at Val(GTC)-146-Phe(TTC), one had a mutation at Ile(ATC)-572-Phe(TTC) in the cluster II region while the fourth isolate was devoid of mutations in either the RRDR or cluster II regions [19,20]. The majority of the mutations (144, 68.2%) were directly identified by three mutation probes (M1, M2 and M3), and the detection rate went up to 77.7% (164) if missing wild type probe signals included. Mutation alone in codon 531 was detected in 116 (55.0%) isolates by mutation probe and in 122 isolates (57.8%) when missing wild type probe signals included (Table 2). DNA sequencing of RRDR showed the mutations in codon 526 were also common in this isolate collection with 37 isolates showing mutations in that codon (Table 2).

**Table 1:** Rifampicin susceptibilities in correlation with the REBA MTB-Rifa® assay on clinical isolates and sputum samples

		**Phenotypic DST**		**Sensitivity**	**Specificity**
		**RIF susceptible**	**RIF resistant**		
Clinical isolates	REBA MTB-Rifa® Assay No mutations in RRDR*	277	4	98.1% (211/215)	100% (277/277)
	REBA MTB-Rifa® Assay Mutations in RRDR*	0	211		
Sputum samples	REBA MTB-Rifa® Assay No mutations in RRDR*	132	0	100% (96/96)	100% (132/132)
	REBA MTB-Rifa® Assay Mutations in RRDR*	0	96		

*RRDR Rifampicin resistance determining regions; *RIF* Rifampicin; *DST* Drug susceptibility testing.

**Table 2:** ***rpoB *mutations in *M. tuberculosis *from South Korea**

**Sequencing**	**REBA MTB-Rifa® assay**	**Sputum**	**Isolates**	**Total**
533CTG-CCG	533CTG-CCG, ∆530-534	8	8	16
531TCG-TTG	531TCG-TTG, ∆530-534	59	115	174
531TCG-TGG	∆530-534	1	3	4
**531TCG-TGC**	∆530-534	1	1	2
531TCG-CAG	∆530-534	0	2	2
526CAC-TAC	∆524-529	4	14	18
526CAC-GAC	∆524-529	4	9	13
526CAC-CTC	∆524-529	2	4	6
526CAC-CCC	∆524-529	1	1	2
526CAC-CGC	∆524-529	1	2	3
526CAC-AAC	∆524-529	0	1	1
526CAC-TGC	∆524-529	0	3	3
526CAC-GGC	∆524-529	0	1	1
526CAC-TCC	∆524-529	0	1	1
522TCG-TGG	∆520-524	0	1	1
518AAC deletion	∆515-520	0	2	2
516GAC-GTC	516GAC-GTC, ∆515-520	6	18	24
516GAC-TAC	∆515-520	3	6	9
516GAC-AAC	∆515-520	0	1	1
**513-514 CAA insertion**	∆509-514	0	1	1
513-516 deletion	∆509-514, ∆515-520	1	1	2
**514TTC-ATC**	∆509-514	1	0	1
513CAA-AAA	∆509-514	1	3	4
513CAA-CTA	∆509-514	1	2	3
511CTG-CCG	∆509-514	0	3	3
511CTG-CCG, 516GAC-GGC	∆509-514, ∆515-520	1	0	1
510CAG-CAC, 511CTG-CCG	∆509-514	1	0	1
531TCG-TTG, 516GAC-TAC	531TCG-TTG, ∆530-534, ∆515-520	0	1	1
533CTG-CCG, 516GAC-GGC	533CTG-CCG, ∆530-534, ∆515-520	0	2	2
526CAC-AAC, 516GAC-AAC	∆524-529, ∆515-520	0	1	1
523GGG-GAG, 513CAA-CTA	∆520-524, ∆509-514	0	1	1
518AAC-CAC, 516GAC-GGC	∆515-520	0	1	1
516GAC-GGC, 515ATG-GTG	∆515-520	0	1	1
516GAC-GTC, 513CAA-GAA	∆515-520, ∆509-514	0	1	1
Total		96	211	307

Bold texts are newly identified mutations in this study; ∆, missing wild type probe signal.

### Detection of rifampicin resistance with the REBA MTB-Rifa® directly on smear positive/culture positive sputum samples

The REBA MTB-Rifa® assay was applied directly to 228 smear positive sputum samples which were culture positive, and polymorphisms were detected in 96 of the 228 samples while wild type alleles were identified in the remaining 132 samples. The REBA MTB-Rifa® assay results were compared to phenotypic DST results as well as DNA sequencing of the RRDR of cultured organisms from the respective sputum samples (Data not shown). The sensitivity was 96/96 (100%; 95% CI 96.2, 100) and the specificity was 132/132 (100%; 95% CI 97.2, 100), respectively (Table 1). The majority of the mutations were detected as missing wild type signals (92/96, 95.8%) and mutation probe could detect specific mutations directly in 73 out of 96 (76.0%) sputum samples.

### Distribution of rifampicin resistance alleles in the r*poB* protein

**Figure 2:**
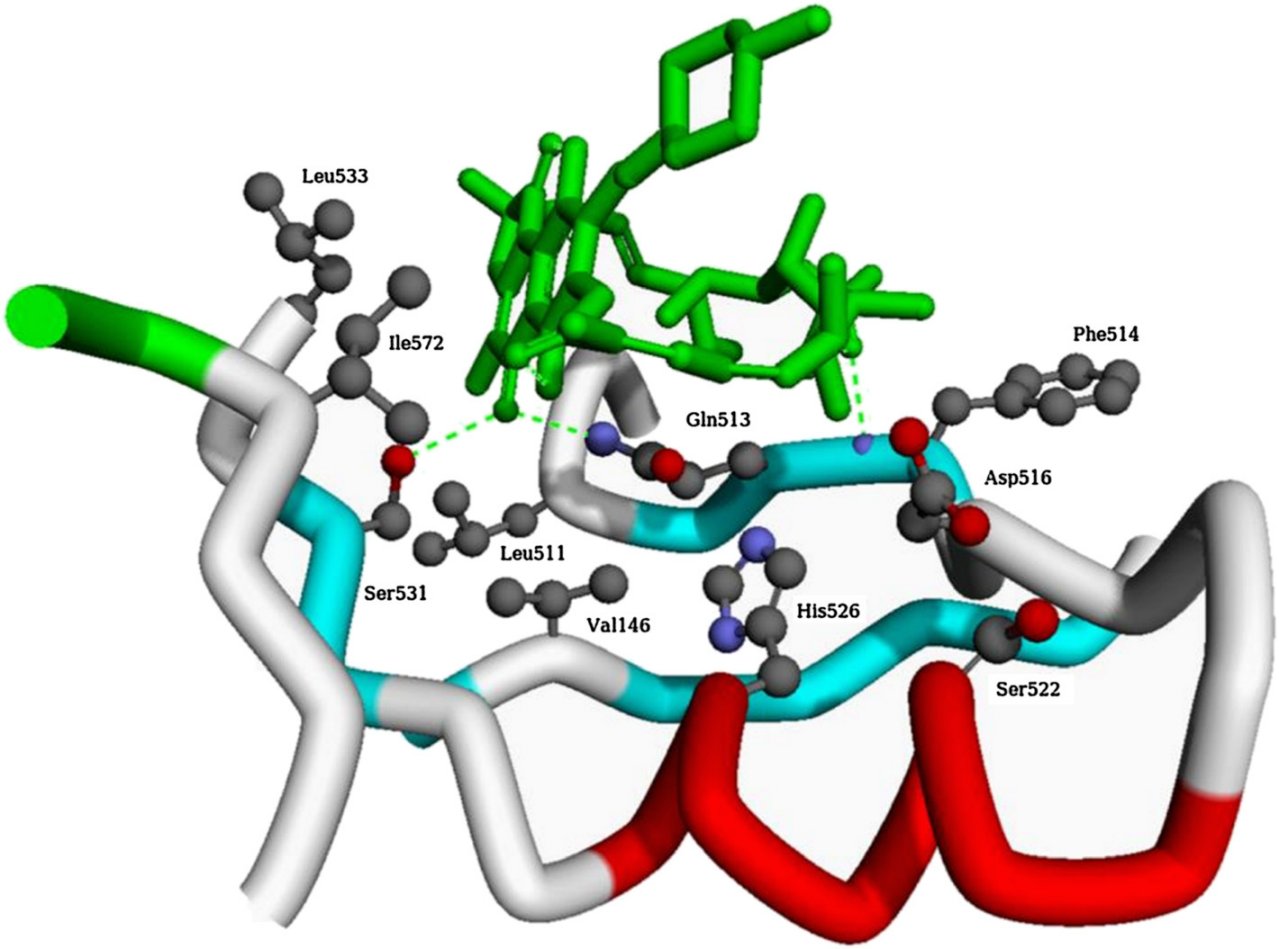
**Representation of the RIF binding site region of RpoB and the 10 sites in which mutations were observed in this study.** The green dotted lines depict hydrogen bonds anchoring the RIF molecule to RpoB. The numbering corresponds to the numbering of the *E. coli* protein but the structure corresponds to the RpoB from *Thermus aquaticus* deposited in PubMed database as 1I6V [32].

Together with isolates and sputum samples, 307 mutations representing 43 different type of mutations in the RRDR of RpoB protein were identified by sequencing in this study, which include 10 double mutations, 2 deletions and a insertion (Table 2). Mutations were located in several regions and some of the regions show higher mutation frequencies than other regions (Figure 2). The codon which most frequently involved in mutations were codon 531 (183, 59.6%), codon 526 (49, 16.0%), codon 516 (42, 13.7%), and codon 533 (18, 5.9%). The most common mutation detected was the Ser(TCG)-531-Leu(TTG), which was seen in 175 (57.0%) of the 307 RIF resistant strains. The second most dominant mutation was observed at Asp(GAC)-516-Val(GTC) (25, 8.1%), followed by His(CAC)-526-Tyr(TAC) (18, 5.9%) (Figure 1).

In addition, three novel mutations were also identified in this study. A mutation from Ser(TCG) to Cys(TGC) at codon 531, a mutation from Phe(TTC) to Ile(ATC) at codon 514 and insertion of CAA between codons 513 and 514 (MIC 12 μg/mL) to the best our knowledge have not been reported hitherto.

## Discussion

This is the first study to validate the REBA MTB-Rifa® assay for the rapid detection of RIF resistance. The study used a large sample size that included both of clinical isolates and smear-positive/culture positive sputum samples (Figure 1)(Table 1). It identified all the RIF resistant sputum samples successfully, and 98.1% (211/215) of all phenotypic RIF resistant *M. tuberculosis* isolates. The sensitivity for the detection of RIF resistance for clinical isolates in this study is slightly higher than in other reports [21,22] and barely lower than the maximum possible detection rate.

It is worth noting that the 98.1% RIF resistant isolates and 100% of smear-positive/culture positive sputum samples with RIF resistance in this study harbored mutations in the RRDR of the *rpoB* gene. Several other reports have shown that about 95% of RIF resistant strains from different countries harbor mutations in the RRDR of the *rpoB* gene. This assay differs from other LPAs in that it took a wild type probe [524-529(WT4)] instead of two mutant probes 526CAC-TAC and 526CAC-GAC, because this codon is very polymorphic [23], In addition this assay also employs a 533CTG-CCG mutant probe instead of a wild type probe, which confers low-level but potentially clinically important RIF resistance that is often missed by phenotypic DST.

The distribution of the mutations observed in this study was generally similar to those reported from other Asian countries [24]. However, the frequency of Ser-531, Asp-516 and double mutations was higher than in Europe, Africa, and the United States [25,26], and the mutation frequency of His-526 was lower than reported in those regions. This suggests that mutational frequency at a specific genomic location can be different possibly as a consequence of strain-specific genetic differences. However, as shown in two reports [24,25], racial factors might also result in different mutation frequencies at specific genomic locations [27,28]. The unique mutations described in this study might be due to racial differences, drug regimens that were taken or treatment compliance of patients as alluded to above [29-31]. Amongst double mutations, newly identified Asp(GAC) to Val(GTC) at codon 516 and Gln(CAA) to Glu(GAA) at codon 513 showed high level phenotypic RIF resistance (MIC >64 μg/mL).

Even though there were 43 different mutation types identified in RRDR from 307 RIF resistant samples (211 isolates and 96 sputum samples), the majority of mutations (292/307, 95.1%) were located in only 4 codons; 516, 326, 531 and 533 in both the banked isolates and the recent sputum samples (Table 2). Some mutations were found only in association with other mutations (codon 510 CAG-CAC; codon 513 CAA-CTA; codon 513 CAA-GAA; codon 515 ATG-GTG; codon 516 GAC-GGC; codon 518 AAC-CAC; codon 523 GGG-GAG) with very low frequency (1–3 samples). There were 4 RIF resistant isolates without any mutations in the RRDR and sequencing analysis were done to identify other mutations in the cluster II region of *rpoB* gene. Mutation at Val(GTC)-146-Phe(TTC) was observed in 2 (0.9%) of these isolates, and this mutation was previously reported in about 1% of RIF resistant isolates, and the level of RIF resistance in this study was as high as in the previous report (MIC 8 μg/mL) [20]. We also observed a mutation at Ile(ATC)-572-Phe(TTC) in an isolate (Figure 1). The remaining isolate did not have any mutations in either of RRDR or cluster II region but nonetheless showed high level of resistance (MIC >64 μg/mL) [19,20]. The failure to detect mutations in cluster II region of RIF resistant isolates, or the cluster II region by the REBA MTB-Rifa® assay suggest that incorporation of the respective mutations in future LPA version would improve the diagnostic accuracy of such tests.

Nearly all of the observed mutations lie within the RIF binding site of the RpoB protein, if not within the canonical RRDR. The two most commonly observed mutations were Ser-531, which is directly involved in hydrogen bonding to the napthol ring and His-526 (Figure 2), which may interact with Asp-516 to form a more extended hydrogen-bonding interaction. The other observed mutations within the RRDR have been reported previously and all participate in direct interaction with various parts of the RIF molecule. Val-146 lies at the bottom of the pocket and, despite being far away in primary sequence, is only 5 Å from the napthol core thereby underscoring its importance. Ile-572 has not been reported in clinical isolates resistant to RIF but it has been noted previously that this residue forms part of a cluster of hydrophobic residues that define one face of the binding pocket and this residue lies less than 4 Å away from the naphthalene ring system [32]. Mutation of this residue to Phe would therefore very likely sterically preclude RIF binding. These results suggest that to achieve 100% coverage of all possible RIF resistance-conferring mutations it may be necessary to include up to a total of 10 additional alleles based upon the RIF binding site.

The majority of the RIF resistant isolates and clinical samples in this study were also MDR-TB suggesting that RIF resistance may not only be a suitable surrogate marker of MDR-TB in high TB incidence settings as suggested by the WHO but in medium TB incidence settings like South Korea as well. A limitation of this study is that the REBA MTB-Rifa® assay was not applied to sputum smear negative cases. This is primarily because our recruitment site predominantly treats confirmed TB cases in smear positive patients. The samples used in this study, therefore may not be representative of the entire South Korean TB population but are a fair representation of the difficult-to-treat TB cases being seen in the national reference hospitals.

## Conclusions

The findings reported herein support the utility of the REBA MTB-Rifa® assay as an alternative tool for the reliable rapid detection of RIF resistance on clinical isolates and smear positive sputum samples in South Korea, and likely in the entire Korean peninsula and surrounding regions as well. Since most (92.1%) of RIF resistant strains in this study were also resistant to INH, the results suggest that RIF resistance may also be a suitable surrogate marker of MDR-TB in a setting of moderate TB incidence such as Korea.

## Abbreviations

TB: Tuberculosis; RIF: Rifampicin; INH: Isoniazid; MDR: Multidrug-resistance; LPA: Line probe assay; REBA: Reverse blot hybridization assay; DST: Drug-susceptibility testing; RRDR: Rifampicin resistance-determining region; XDR-TB: Extensively drug-resistant TB; EMB: Ethambutol; PZA: Pyrazinamide; NMH: Masan national hospital; ZN: *Ziehl-Neelsen*; PCR: polymerase chain reaction; SSPE: Saline-sodium phosphate- ethylenediaminetetraacetic acid; SDS: Sodium dodecyl sulfate.

## Competing interests

The authors declare that they have no competing interests.

## Authors’ contributions

EJC, HKK, JIL participated in the design of the study, acquired and analyzed the data. JSL participated in the design of the study, analyzed, interpreted the data, drafted the manuscript and critically revised the manuscript. ICS participated in interpreting the data and critically revised the manuscript. MSL, SHH, HSK, DSJ, CTK, SNC, LEV and CEB participated in the design of the study, analyzed, interpreted the data and critically revised the manuscript. All authors read and approved the final manuscript.

## Pre-publication history

The pre-publication history for this paper can be accessed here:

http://www.biomedcentral.com/1471-2334/13/478/prepub
